# Potential Polyunsaturated Aldehydes in the Strait of Gibraltar under Two Tidal Regimes

**DOI:** 10.3390/md12031438

**Published:** 2014-03-13

**Authors:** Soledad Morillo-García, Nerea Valcárcel-Pérez, Andrés Cózar, María J. Ortega, Diego Macías, Eduardo Ramírez-Romero, Carlos M. García, Fidel Echevarría, Ana Bartual

**Affiliations:** 1 Department of Biology, University of Cádiz, Avda. República Saharaui s/n., Puerto Real, Cádiz 11510, Spain; E-Mails: soledad.morillo@uca.es (S.M.-G.); nerea.valcarcel@uca.es (N.V.-P.); andres.cozar@uca.es (A.C.); eduardo.ramirez@uca.es (E.R.-R.); carlos.garcia@uca.es (C.M.G.); fidel.echevarria@uca.es (F.E.); 2 Department of Organic Chemistry, University of Cádiz, Avda. República Saharaui s/n., Puerto Real, Cádiz 11510, Spain; E-Mail: mariajesus.ortega@uca.es; 3 Institute for Environment and Sustainability, Joint Research Center, European Commission, Via E. Fermi, 2147, Ispra, Varese 21021, Italy; E-Mail: diego.macias-moy@jrc.ec.europa.eu

**Keywords:** polyunsaturated aldehydes, diatoms, physiological stress, Strait of Gibraltar, heptadienal, octadienal, decadienal

## Abstract

Diatoms, a major component of the large-sized phytoplankton, are able to produce and release polyunsaturated aldehydes after cell disruption (potential PUAs or *p*PUA). These organisms are dominant in the large phytoplankton fraction (>10 µm) in the Strait of Gibraltar, the only connection between the Mediterranean Sea and the Atlantic Ocean. In this area, the hydrodynamics exerts a strong control on the composition and physiological state of the phytoplankton. This environment offers a great opportunity to analyze and compare the little known distribution of larger sized PUA producers in nature and, moreover, to study how environmental variables could affect the ranges and potential distribution of these compounds. Our results showed that, at both tidal regimes studied (Spring and Neap tides), diatoms in the Strait of Gibraltar are able to produce three aldehydes: Heptadienal, Octadienal and Decadienal, with a significant dominance of Decadienal production. The PUA released by mechanical cell disruption of large-sized collected cells (*p*PUA) ranged from 0.01 to 12.3 pmol from cells in 1 L, and from 0.1 to 9.8 fmol cell^−1^. Tidal regime affected the abundance, distribution and the level of physiological stress of diatoms in the Strait. During Spring tides, diatoms were more abundant, usually grouped nearer the coastal basin and showed less physiological stress than during Neap tides. Our results suggest a significant general increase in the *p*PUA productivity with increasing physiological stress for the cell also significantly associated to low nitrate availability.

## 1. Introduction

Diatoms often dominate the fraction of the large-sized phytoplankton in the ocean. The reason of this ecological success still constitutes a matter of discussion and diverse mechanisms have been proposed to explain its widespread presence in the ocean [1,2,3]. Among these mechanisms, there are evidences that diatoms have a chemical defense system, which can potentially affect both predators [4,5] and competitors [6,7]. It has been demonstrated that different diatoms species are able to produce a wide range of secondary metabolites belonging to the oxylipins family [8]. These bioactive compounds include volatile polyunsaturated aldehydes (here abbreviated as PUAs), such as Heptadienal (HEPTA), Octadienal (OCTA), Octatrienal, Decadienal (DECA) and Decatrienal [4,9,10]. The production and release of PUAs occur by lipoxygenase-mediated degradation of free polyunsaturated fatty acids (PUFAs) after cell damage [11]. Pohnert [5] proposed that these compounds could take part in the chemical defense system of diatoms, by interfering with the reproductive success of grazers*.* Experimental research has shown that PUAs production in diatoms is linked to the physiological and environmental conditions prevailing during growth [12,13,14]. However, information regarding PUAs production in nature is still scarce [15]. There are oceanic areas where the microplanktonic fraction is usually dominated by diatoms and constitute valuable natural laboratories for the study of the ranges of variability of these compounds in natural conditions. This is the case of the Strait of Gibraltar, where diatoms represent a dominant phytoplankton group [16].

In the present study, we examine the PUA productivity of the large-sized phytoplankton in the Strait of Gibraltar. This area is the only connection between the Mediterranean Sea and the Atlantic Ocean. The water circulation through the Strait is characterized by a two-layer system [17] with an upper Atlantic layer inflowing into the Mediterranean Sea, and Mediterranean water outflowing at deep [18]. Phytoplankton cells undergo wide changes in water turbulence and nutrient availability in this highly dynamic system, mainly due to intense mixing processes and nutrient circulation linked to the tidal cycles. At seasonal scale, the intense mixing linked to spring tide regime is usually associated to higher nutrient availability [19,20]. In this study, we have sampled the spatial distribution of PUAs producers under two different tidal regimes, Spring Tides (ST) and Neap Tides (NT), which must be associated with different environmental conditions (e.g., turbulent kinetic energy, light and nutrient availability) [19,20,21]. The concentrations of PUAs were measured after the artificial cell disruption in natural assemblages of large-sized phytoplankton (>10 µm). In addition, we monitored the changes in nutrient availability, phytoplankton composition and percentage of active chlorophyll in cells (as indicator of physiological stress in phytoplankton [22]) in order to find insight on the environmental control of PUAs production in nature.

## 2. Results and Discussion

### 2.1. Plankton Distribution: Abundance, Biovolume and General Characterization

**Figure 1:**
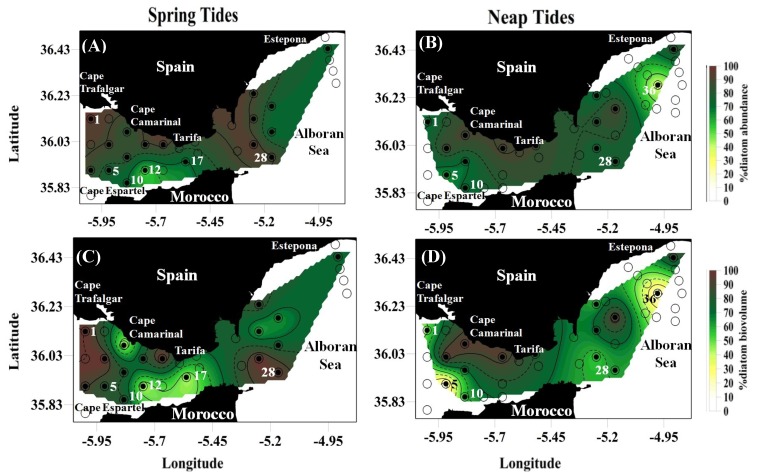
Spatial distribution of diatoms in the Strait of Gibraltar expressed as percentage of abundance (**A**,**B**) and percentage of biovolume (**C**,**D**) with respect to total large sized phytoplankton under two tidal regimes (Spring tides and Neap tides). The open circles indicate all sampling stations. The closed circles indicate stations where phytoplankton samples were analyzed by FlowCam methodology (See Experimental Section for details). For clarity, only stations directly named in the text have been numbered. All stations are numbered at the picture in the Experimental Section. Some geographical areas are also indicated.

The fraction of larger sized phytoplankton (>10 µm) was dominated by diatoms, as expected (Table 1, Figure 1). Diatoms represented more than 70% of phytoplankton cell abundance at both tidal regimes (Table 1), followed by a 15%–20% of dinoflagellates and lower percentages of coccolitophorids and silicoflagellates (0.03% and 0.8%, respectively). Diatom cell abundance varied from a minimum of 0.06 × 10^3^ cell L^−1^ (St 28 under Neap tide regime) to a maximum of 50.9 × 10^3^ cell L^−1^ (St 1, under Spring tide regimes). The obtained ranges of cell abundance were similar to that obtained in this area by other authors (0–11 × 10^3^ cell L^−1^) [16]. Cell abundance never reached levels as those usually found in diatoms blooming conditions (10^6^ cell L^−1^), offering an interesting background for *p*PUA analyses since these compounds have been usually quantified either on experimental cultures [9,23] or natural blooming conditions [15] but never before under non blooming oceanic areas.

Diatoms were found near northern coasts during Spring Tides (ST), especially near Cape Trafalgar, but had a more disperse pattern during Neap Tides (NT) (Figure 1A,B). Only some stations showed high percentage of dinoflagellates, as St 36 during Neap Tide cycles (Supplementary Table S1). This particular site is located in the Mediterranean sector of the Strait, in an area where upwellings frequently occurs. The abundance of grazers, mostly belonging to copepods and tintinnids, was considerably lower than phytoplankton (<3%) (Table 1), reaching a maximum at St 5 during the NT cycle (13.4%) (Supplementary Table S1).

**Table 1:** Averaged percentage of cell abundance and cell biovolume (mean ± standard deviation, SD) and ranges (minimum-maximum) for diatoms, dinoflagellates and grazers (copedods and tintinnids) in relation to the total large-sized plankton (>10 µm), under Spring and Neap tidal regimes.

		% Abundance of the total large-sized plankton		% Biovolume of the total large-sized plankton	
		mean ± SD	range	mean ± SD	range
**Spring Tides**	**Diatoms**	75.0 ± 14.5	51.1–95.9	63.4 ± 27.7	19.5–99.9
	**Dinoflagellates**	13.9 ± 8.5	2.0–34.4	13.4 ± 11.9	0–42.8
	**Copepods + Tintinnids**	1.5 ± 1.3	0–4.7	16.1 ± 15.5	0–46.9
**Neap Tides**	**Diatoms**	69.9 ± 17.4	34.6–90.8	44.8 ± 29.3	1.3–93.0
	**Dinoflagellates**	21.5 ± 13.0	7.7–59.7	18.9 ± 13.6	1.9–49.9
	**Copepods + Tintinnids**	2.7 ± 3.0	0.3–13.4	25.6 ± 9.0	1.4–52.5

The differences between tidal regimes were much more evident when the plankton concentrations are expressed in bio-volume units, µm^−3^ L^−1^ (Figure 1C,D and Table 1). In biovolume, the averaged percentage of diatoms becomes significantly higher (65%–99%) in ST (*p*-value < 0.05). Most of the stations (60%) showed diatom predominance in ST. In NT, diatoms were dominant in 40% of stations sampled. These ranges of diatom biovolume (Supplementary Tables S1 and S2) are similar to that obtained in previous studies [16].

Diatoms were generally dominated by helical chains, including species as *Guinardia striata* or *Chaetoceros debilis* (Table 2, Supplementary Information of FlowCAM images). Three stations (St 10, St 12 and St 17 in Figure 1) showed a predominance of the linear chains of small cells (e.g., *Chaetoceros* sp., *Skeletonema* sp.) and large cells (e.g., *Rhizosolenia* sp., *Proboscia* sp.) during ST. In these three stations, diatoms were the dominant group of plankton by cell abundance. However, grazers were the most important in biomass (measured as biovolume) (Supplementary Table S1). The predominace of large phytoplankton could be explained from a selective predation of grazers over small diatoms and non chain forming species, while large diatoms would accumulate in refuge sizes where the grazing pressure declines.

**Table 2:** Averaged percentage of cell abundance and cell biovolume (mean ± standard deviation, SD) for different groups of diatom relative to the total abundance of large-sized phytoplankton fraction (autotrophic fraction >10 µm), under Spring and Neap tidal regimes. The main characteristics of the diatom groups are described in the Experimental Section and photographs are available in the Supplementary Information.

		% Abundance of the total large-sized plankton	% Biovolume of the total large-sized plankton
		mean ± SD	mean ± SD
**Spring Tides**	**Diatoms**	80.9 ± 12.2	77.4 ± 19.9
	**Centric singles diatoms**	5.5 ± 6.0	2.3 ± 4.4
	**Pennate diatoms**	1.4 ± 2.7	1.8 ± 4.1
	**Lineal small cells chains**	11.1 ± 16.0	2.3 ± 6.4
	**Lineal big cells chains**	15.4 ± 6.2	11.6 ± 10.7
	**Helical chains**	62.0 ± 23.7	77.0 ± 22.4
	**Other diatoms**	4.4 ± 3.4	4.7 ± 6.7
**Neap Tides**	**Diatoms**	74.3 ± 15.5	62.3 ± 29.3
	**Centric singles diatoms**	4.7 ± 5.8	7.6 ± 12.6
	**Pennate diatoms**	4.8 ± 15.9	8.5 ± 23.2
	**Lineal small cells chains**	9.9 ± 10.5	1.1 ± 1.6
	**Lineal big cells chains**	8.3 ± 15.3	5.8 ± 7.6
	**Helical chains**	68.7 ± 27.8	68.0 ± 31.3
	**Other diatoms**	3.3 ± 3.9	8.7 ± 11.8

### 2.2. Total Chlorophyll, Fractionated Chlorophyll and Active Chlorophyll

Averaged values of Total Chlorophyll (TChla) and fractionated chlorophyll (FChla > 20 µm) were significantly higher during Spring Tide cycles (*p*-value < 0.01) (Table 3). Maximum values of both variables were measured near the coast, as described in [20]. The contribution of FChla to TChla also was significantly different in the two tidal regimes. During ST, 70% of TChla variability was explained by FChla (*R*^2^: 0.70), and only a 30% during NT (Supplementary Figure S1). These results agree with the significantly higher diatom biovolume found during ST (Table 1).

**Table 3:** Averaged concentrations (mean ± standard deviation, SD) and ranges (minimum-maximum) of total Chlorophyll a (TChla, mg m^−3^), fractionated chlorophyll >20 µm (FChla, mg m^−3^) and active chlorophyll (AChla, percentage of TChla) measured at the Strait of Gibraltar under Spring and Neap tidal regimes. Values for individual stations are detailed in Supplementary Tables S1 and S2.

	Spring Tides		Neap Tides	
	mean ± SD	range	mean ± SD	range
**TChla**	1.3 ± 1.2	0.1–4.2	0.7 ± 0.4	0.2–1.8
**FChla**	0.1 ± 0.1	0.3–2.0	0.1 ± 0.09	0–0.2
**AChla**	26.4 ± 18.9	0–65.0	8.6 ± 13.5	0–36.3

The percentage of active chlorophyll (AChla) in relation to TChla can be used as indicator of the physiological state of photosynthetic cells [22]. Lower values of AChla suggest strong physiological stress and low photosynthetic efficiency, usually associated to senescent cells. Averaged percentage of AChla were significantly lower during NT (8.6% ± 13.5%) compared with ST (26.4% ± 18.9%) (*p*-value < 0.001) (Table 3). In addition, 29% of stations sampled in ST showed values of 0% of AChla, whereas this percentage rose up to 59% during NT (Supplementary Table S1). These results suggest that algal cells underwent relatively strong physiological stress in most of the stations during NT regime.

### 2.3. Nutrients

As expected, average concentration of nitrate and phosphate were higher during ST (Table 4). Phosphate was the minoritary nutrient at both tidal regimes, with concentrations equivalent to previously found in the region [24]. Making an stoichiometric approach on the average concentrations, N:P ratio was far above the Redfield ratio (N:P = 16) [25] for both tidal situations (N:P = 36 for ST and N:P = 24 for NT) (Table 4) coinciding also with low mean phosphate values (0.05 and 0.03 µM) (Table 4). In fact, maximum concentration of phosphate during ST was moderate (0.49 µM) and levels of phosphate were undetectable in most of the stations (60%–80%) at both tidal regimes (see Supplementary Tables S1 and S2). Therefore, a general phosphorus limitation can be inferred for the sampled region, with local inputs in some local areas. Regarding nitrate and silicate, low average levels were also observed in both tidal regimes. Considering the ranges of semi-saturation constant (Ks) for nitrate and phosphate for most diatoms (Ks NO_3_ = 7.29 ± 8.77 µM, Ks PO_4_ = 1.59 ± 1.81 µM; [26]) and for silicate (Ks SiO_4_: 0.8 to 3.37 µM [27,28]), some local areas may also show deficiencies on nitrate and silicate.

**Table 4:** Averaged concentrations (mean ± standard deviation, SD) and ranges (minimum-maximum) of dissolved Silicate (µM), Nitrate (µM) and Phosphate (µM) measured at Strait of Gibraltar under ST and NT regimes. Data are grouped by geographical sectors defining by longitudes: Atlantic sector, Mediterranean sector and Estepona upwelling (north-eastern coast). Values for individual stations are detailed in Supplementary Tables S1 and S2. n.d. undetectable levels.

		Mean ± SD	Range	Atlantic (−6,−5.6)	Mediterranean (−5.59,−5.16)	Estepona (−5.20,−4.87)
**Spring Tides**	**Silicate**	2.2 ± 1.8	0.6–9.9	3.0 ± 2.2	1.3 ± 0.6	1.6 ± 1.1
	**Nitrate**	1.8 ± 1.6	0–8.4	2.4 ± 2.0	1.4 ± 0.9	0.6 ± 0.7
	**Phosphate**	0.05 ± 0.1	n.d.–0.04	0.06 ± 0.1	0.06 ± 0.1	n.d.
**Neap Tides**	**Silicate**	4.0 ± 6.1	0.5–27.1	1.3 ± 0.3	2.2 ± 1.0	7.6 ± 8.9
	**Nitrate**	0.72 ± 0.9	n.d.–3.3	0.2 ± 0.3	1.1 ± 1.1	0.1 ± 0.2
	**Phosphate**	0.03 ± 0.05	n.d.–0.21	0.03 ± 0.06	0.03 ± 0.05	0.008 ± 0.02

Examining the nutrient concentration along the Strait, we find a decreasing gradient from western to eastern sector of the Strait during ST and an opposite trend during NT (Table 4). Nutrient dynamics in the Strait of Gibraltar is strongly modulated by tide-induced mixing due to the intense two-layer water circulation. These mixing processes may results, especially in ST, in inputs of nutrients to the photic layer [19,21] as it can be inferred from our results.

### 2.4. pPUA Ranges and Spatial Distribution

The collected microplankters, mainly diatoms as explained in the above section, produced three PUAs by artificial cell disruption: Heptadienal (HEPTA), Octadienal (OCTA) and Decadienal (DECA). Other PUAs commonly released by diatom strains in unialgal cultures, as Decatrienal or Octatrienal, were not detected in our samples, although this does not discard the possibility that some diatoms species sampled could produce other PUAs at non detectable levels. Concentrations of total *p*PUA (as a summation of HEPTA, OCTA and DECA) measured for the whole of the sampled stations, ranged between 0 and 9.8 pmol from cells in 1 L (Table 5). This range is much lower than nM levels reported by [15] in the Adriatic Sea. Two main reasons could explain such differences. First, here we have quantified the PUA released by artificial wounding of the larger sized phytoplankton fraction (a mesh diameter of 10 µm), compared with a wider cell size spectra collected by [15] (a mesh diameter used for collecting cells of 1.2 µm). Second, cell densities are three orders of magnitude lower in the Strait of Gibraltar (Supplementary Tables S1 and S2) compared with the Adriatic Sea, where samples were taken after a bloom event of *Skeletonema marinoi* [15]. For a correct comparison of *p*PUA levels, these should be normalized by cell density. Then, the obtained values in phytoplankton assemblages from the Strait of Gibraltar ranged from 0.04 to 9.81 fmol cell^−1^ in Spring Tides and from 0.1 to 7.3 fmol cell^−1^ in Neap Tides, which are in the range previously measured for diatoms in cultures [23,29,30]. Our concentrations are also coherent with the 13 fmol cell^−1^ obtained from [15].

**Table 5:** Concentrations of total polyunsaturated aldehydes (*p*PUA = HEPTA + OCTA + DECA) measured after artificial cell disruption of plankton cells contained in the samples (pmol from cells from 1 L) and Concentrations of total polyunsaturated aldehydes (*p*PUA = HEPTA + OCTA + DECA) per cells from 1 L (fmol cell^−1^) under Spring and Neap tidal regimes. Data are expressed as averaged concentrations (mean ± standard deviation, SD) and ranges (minimum-maximum). Values for sampling sites are shown in Supplementary Tables S1 and S2.

		*p*mol from cells from 1 L		fmol cell^−1^	
		mean ± SD	range	mean ± SD	range
**Spring Tides**	**HEPTA**	0.7 ± 0.5	0.02–1.98	0.3 ± 0.6	0–2.7
	**OCTA **	0.5 ± 0.7	0–3.10	0.2 ± 0.5	0–2.3
	**DECA**	1.6 ± 1.9	0.01–8.74	0.8 ± 1.1	0–4.7
	**Total PUAs**	2.9 ± 3.0	0.05–12.3	1.4 ± 2.3	0–9.8
**Neap Tides**	**HEPTA **	0.8 ± 0.6	0–3.10	0.7 ± 1.3	0–5.3
	**OCTA**	0.2 ± 0.4	0-1.76	0.2 ± 0.4	0-1.3
	**DECA**	1.3 ± 1.4	0.01–5.7	0.9 ± 0.9	0–2.6
	**Total PUAs**	2.3 ± 2.4	0.04–9.81	1.9 ± 2.1	0.1–7.3

During the last two decades, several researchers have described different phytoplankton species as producers of HEPTA, OCTA and DECA in experimental cultures [4,9,10,31]. In nature, diatoms as *Skeletonema marinoi* have shown to be important producers of HEPTA and OCTA in oceanic coastal areas when blooms take place [15] and other non diatom genera as *Phaeocystis* have been reported to produce DECA [23] during blooming events. Recently, a significant presence of these three PUAs has been described in open oceanic areas [32].

**Figure 2:**
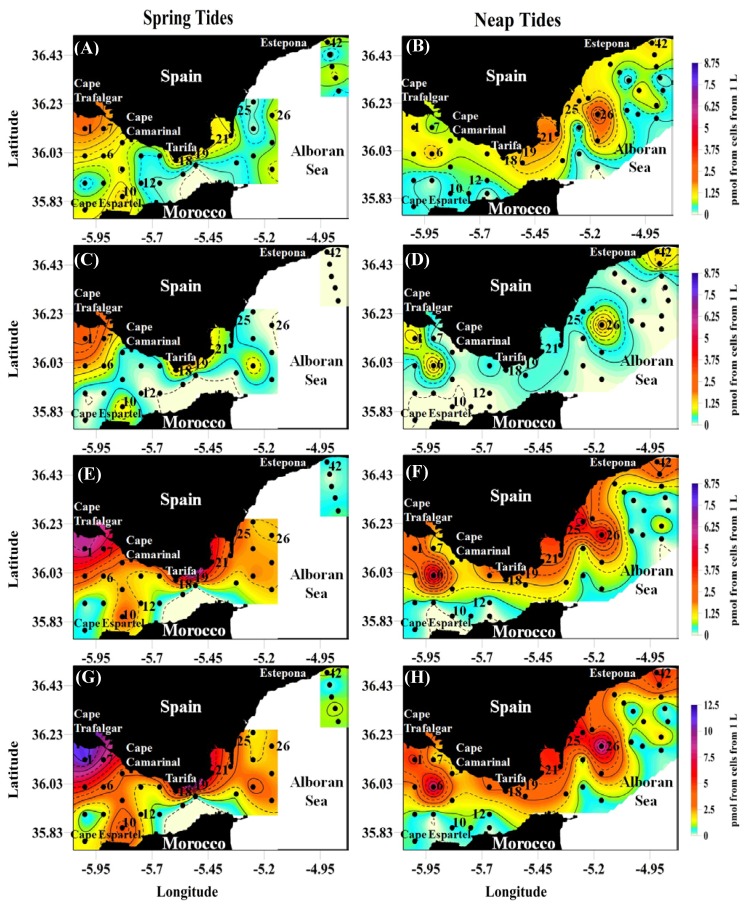
Spatial distribution of *p*PUA (**A**,**B**) Heptadienal; (**C**,**D**) Octadienal and (**E**,**F**) Decadienal; (**G**,**H**) Total PUA as summation of three aldehydes from large size phytoplankton collected at 25 m depth in waters of the Strait of Gibraltar, between September and October 2008, for two different tidal regimes (Spring Tides and Neap Tides). Concentrations are expressed in pmol from cells from 1 L. The closed circles indicate the sampling stations. Extrapolation used was Krigging. To avoid artifacts in extrapolation of the data, white color is used to show non-sampled areas. Note the difference scales in **G** and **H**.

There were no significant differences in the averaged concentration of *p*PUA (DECA + HEPTA + OCTA) for both tidal regimes (Table 5). By aldehydes, DECA was the most abundant in both tidal regimes with a maximum value of 8.74 pmol from cells in 1 L in St 1 and St 19 during ST (Table 5, Figure 2C,E), followed by HEPTA, with maximum values obtained during NT (3.10 pmol from cells from 1 L, St 26) (Table 5, Figure 2A). OCTA was less abundant but reaching higher values during Spring Tide regime. By cell unit, the average value of *p*PUA was slightly higher during NT (Table 5) and HEPTA reached the highest levels. Maximum values of *p*PUA per cell unit were found in stations St 10 and St 12 during ST, corresponding with stations rich in chains of *Skeletonema* species, which is consistent with high production of HEPTA and OCTA by this genus at experimental and natural conditions [15,29].

The spatial distribution of *p*PUA producers found in the Strait of Gibraltar (Figure 2) showed a similar pattern to that obtained for diatom distribution (Figure 1). Stations with maximum values of *p*PUA were coincident with maximum percentage of diatoms in most cases. However, we quantified *p*PUA in all stations (42 stations at each tidal regime) while diatom abundance was restricted to 37 (19 stations during ST and 18 during NT).

During Spring Tide cycles, maximum *p*PUA values were found in the Atlantic sector of the Strait and near to the coast (Figure 2). In the northern coast, maximum levels of HEPTA, OCTA and DECA were found in the region of Cape Trafalgar (St 1 and St 7) and Tarifa (St 18 and St 19). In the Moroccan coast, maximum values were obtained in the stations placed near Cape Espartel (St 10) (Figure 2). In contrast, *p*PUA were widespread during NT regime, with maximum values offshore (St 6, St 26) (Figure 2). Coastal maxima were located in the Mediterranean sector during this tidal regime (St 21, St 25, St 42).

Maximum concentration of diatoms and *p*PUAs were found near the coast during the ST phase, being the continental shelf close to Cape Trafalgar particularly important. This area is characterized by an intense and steady dynamic related to tidal circulation at mesoscale, leading to quasi-permanent nutrient input to surface [33]. This nutrient supply will lead to high values of chlorophyll and high percentage of active cells with some bloom episodes, which have been reported mainly for September [34], and a high productivity.

Considering that diatoms dominate the phytoplankton fraction of the Strait, the relevance of DECA contribution to total *p*PUA compared with OCTA and HEPTA is a non-expected result according to the available bibliography. Although some diatoms species have shown to be DECA producers in culture as *Thalassiosira rotula*, *Chaetoceros compressus*, *Melosira nummuloides* or *Fragilaria* sp. [17], existing studies suggest that most diatoms are not important DECA producers, and HEPTA and OCTA have shown to be the most abundant PUA [29]. In nature, *Skeletonema marinoi* does not produce DECA in natural blooming conditions [15], but some areas rich in PUA producers have been sampled in the Subtropical Atlantic [32]. This result opens new questions regarding the importance of diatoms as DECA producers in nature.

Our taxonomic analyses of the planktonic fraction are not enough to definitively infer species responsible for PUA production, but it allows inferring that diatoms were responsible for that. We have artificially induced the production of PUA by sonication of the collected natural assemblages that would be constituted by several diatom and non diatom species, with attached bacteria, more or less aggregated and with a physiological pre-story. Then, our results indicate that phytoplankton species collected in the Strait during the sampling period, mainly diatoms, had the adequate metabolic machinery to produce these three aldehydes in the exact moment that they were collected. This machinery would include, at least, the polyunsaturated fatty acids that act as PUA precursors (eicosapentanoic acid for HEPTA, Hexadecatrienoic acid for OCTA and Arachidonic acid for DECA) [9,11] and enzymatical resources necessary for lypooxidation once the cell is wounded. Availability of these metabolites would be highly dependent on the physiological state of the cell and the availability of resources necessaries for its synthesis (*i.e.*, nutrients). Cell physiological state can significantly and rapidly change as a response to variable environmental conditions. On the contrary, experimental cultures are usually driven with unialgal and axenic cultures at very stable environmental conditions, defined by stable abiotic (temperature, light, nutrient input, organic matter) and biotic parameters (unialgal cultures usually monoclonal, without pathogens and without competitors). The obtained results in such conditions would give us a very valuable representation of the PUA spectra potentially produced by the studied strain with a standard metabolic profile but it does not necessary resemble the common trend in nature. Several authors have shown certain variability of the lipidic pool as a response to changes in physiological conditions or environmental factors as culture age, nutrient availability or temperature [35]. In addition, toxins and secondary metabolites production of allelopathic species can be affected by physiological conditions [36]. Moreover, Ribalet *et al.* [12,13] demonstrated that the potential for PUA production of *Skeletonema marinoi* increased with age and changes under N and P limitation conditions and propose a direct link between PUA production, physiological stress and nutrient stress. Additionally, the observed variability in metabolites profile at different experimental conditions in the diatom *Skeletonema marinoi* [37] supports the previous hypothesis.

To assess the influence of the environmental and biological variables on *p*PUA and PUA per cell distribution, a principal component analysis (PCA) was carried out. This analysis reduces a large set of variables to a simplified set still containing most of the information. The variables considered has been percentage of active chlorophyll (AChla), fractionated chlorophyll (FChla), silicate, nitrate, phosphate, percentage of diatom biovolume (DTB), percentage of dinoflagellates biovolume (DNB) percentage and percentage of grazers biovolume (GZB) (Table 6). Others variables such as total chlorophyll and the percentage of abundance of diatom (DTA), dinoflagellates (DNA) and grazers (GZA) were excluded from the PCA because there were a high correlation, and it is appropriate use independent variables.

**Table 6:** Loadings of the different principal components (PC1 and PC2) in both tidal regimes. The strong significant loadings (>0.7) are printed in bold. The percentage indicates the contribution of each principal component to the total variance. PCA was performed using the program R [38].

	Spring Tides		Neap Tides	
	PC1 (75.0%)	PC2 (17%)	PC1 (84.0%)	PC2 (8%)
AChla	0.40	**0.88**	0.24	−**0.94**
DTB	**0.80**	−0.35	**0.83**	0.24
DNB	−0.27	<0.01	−0.37	−0.18
GZB	−0.32	0.29	−0.32	0.11
FChla	<0.01	<0.01	<0.01	<0.01

The PCA analysis for ST extracted two main factors that explained 75% and 17% of the total variability of PUA levels (Table 6). The first factor (PC1) was strongly and positively correlated with diatom biovolume (DTB, 0.80) and negatively correlated with grazer biovolume (GZB, −0.32) and the second (PC2) was positively correlated with Active Chorophyll (0.88). PCA for NT showed one factor explaining 84% of the total variability of PUA data, which was positively correlated with diatom biovolume (DTB, 0.83) and negatively with grazers biovolume (GZB, −0.32).

Stations were then ordered following a classification into two main groups at both tidal regimes according to results of PCA: **GROUP 1:** stations with high abundance (82%–95%) of diatoms and high percentage (65%–99%) of diatom biovolume and **GROUP 2:** stations with high abundance (82%–95%) of diatoms but low percentage of diatom biovolume (7%–50%). Stations of each group were ordered in the basis of percentage of active chlorophyll (AChla; CHA in PCA) as indicative of physiological stress: (A) 40%–70% (non stressed cells) and (B) 20%–40% (moderate stressed cells) or 0%–20% (strongly stressed cells) (Table 7).

Under NT regime, diatoms were in all cases under moderate or strong physiological stress obtaining the highest averaged values of PUA per cell, however, under ST, we could found stations were diatoms were under no physiological stress. The causes of this physiological stress are probably diverse, however these results are coherent with lower average concentration of nitrate, silicate and specially, phosphate during NT compared with ST (Table 4). Small diatoms were mainly under stress (no stations on groups 1A and 2A during NT neither at 2A during ST) associated in most stations to higher relative biovolume of grazers (Supplementary Tables S1 and S2), as St36 (Group 2B) where the biovolume of grazers increased to a 63%. In this station, where *Skeletonema* and *Chaetoceros* chains are the dominant group of diatoms, the highest value of *p*PUA per cell (7.3 fmol cell^−1^) mainly HEPTA was found. During ST, also particular stations with low nitrate availability as St9 (Supplementary Table S1) showed higher PUA per cell content.

**Table 7:** Classification of stations sampled according to PCA analyses. *p*PUA: pmol from cells from 1 L; PUA per cell: fmol cell^−1^; AChla: percentage of active Chlorophyll.

Spring Tides		Neap Tides	
**GROUP 1: Stations with High Percentage of Diatom Biovolume (Large Size Cells)**			
*Group 1A No Physiological Stress (AChla 40%–70%) Stations: 1,6,14,23*	*Group 1B Strong and Moderate Physiological Stress (AChla 0%–40%) Stations: 3,5,9,13,25,28*	*Group 1A No Physiological Stress (AChla 40%–70%)*	*Group 1B Strong and Moderate Physiological Stress (AChla 0%–40%) Stations: 6,8,14,19,26,41*
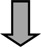	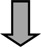	No Stations	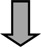
*p*PUA: 1.8–11.4 PUA per cell: 0.1–0.6	*p*PUA: 0.3–3.0 PUA per cell: 0.16–1.43		*p*PUA: 0.05–9.81 PUA per cell: 0.059–4.89
**GROUP 2: Stations with Low percentage of diatom biovolume (small size cells)**			
*Group 2A No Physiological Stress (AChla 40%–70%)*	*Group 2B Strong and Moderate Physiological Stress (20% < AChla < 40%) Stations: 8,10,12,24,26,27,41*	*Group 2A No Physiological Stress (AChla 40%–70%)*	*Group 2B Strong Physiological Stress (AChla 0%–40%) Stations: 1,5,9,10,23,24,25,36*
No Stations	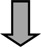	No Stations	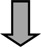
	*p*PUA: 0.05–5.06 PUA per cell: 0.05–9.89		*p*PUA: 0.3–6.8 PUA per cell: 0.34–7.3

**Figure 3:**
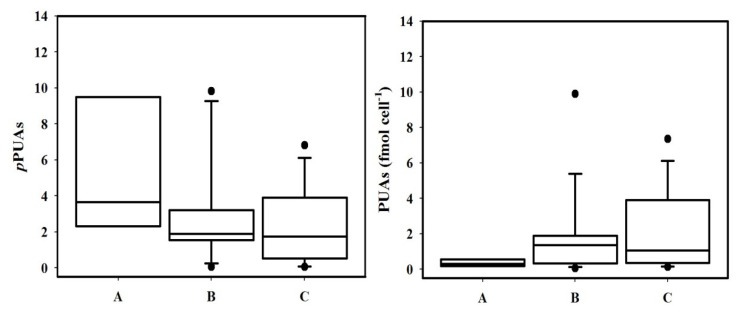
Box-plot of *p*PUA (pmol from cells in 1 L) (**left**) and PUA per cell (fmol cell^−1^) (**right**) grouped by percentage of Active Chlorophyll categories (named A, B and C) as indicative of physiological stress in three categories: A: No physiological stressed cells (40%–70% of ACla), *n* = 4 stations; B: moderate physiological stress (20%–40%); *n* = 16 stations, and C: strongly physiological stressed cells (0%–20%); *n* = 13 stations. *t*-test was used for the comparison of data from the three physiological stress categories.

Grouping PUA concentrations exclusively on the basis of physiological stress (using ACla as stress indicator) and without considering tidal regime or spatial distribution, the relationship between PUA and physiological stress become clearer (Figure 3). PUA per cell increased toward higher stressed cells categories, although with a high range of variability (*p* > 0.05) as expected for nature.

**Figure 4:**
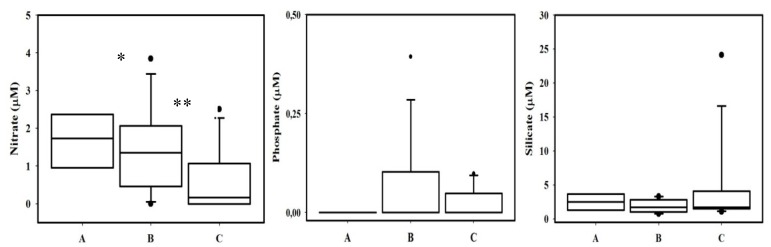
Box plot of dissolved nutrient concentration in stations grouped in three categories (A, B and C) according to the level of physiological stress of collected cells. A: No physiological stressed cells (40%–70% of ACla), *n* = 4 stations; B: moderate physiological stress (20%–40%); *n* = 16 stations, and C: strong physiological stressed cells (0%–20%); *n* = 13 stations. * denotes significantly different averages (*t*-test was used for comparison, * *p* < 0.05, ** *p* < 0.01).

Ranges of PUA per cell obtained for each category fit well with trends obtained by [12,13] in experimental cultures for the diatom *Skeletonema marinoi*, where PUA per cell increased from 1.23 ± 0.37 fmol cell^−1^ under nutrient repleted conditions (no stressed cells) to 7.49 ± 0.08 fmol cell^−1^ under P-limited and 5.9 ± 0.94 fmol cell^−1^ under N-limited (stressed cells). As shown in Figure 4, a significant (*p* < 0.05) decrease of dissolved nitrate was associated to the increasing physiological stress, with low average phosphate concentrations. At least, nitrate and probably phosphate stress can be inferred for these results.

Our results support the hypothesis that physiological stressed diatoms could release higher amounts of PUAs in nature. Such trend has been also observed in experimental cultures [12,13], but the goal here is that it has been described for natural assemblages. Here we have only considered the larger fraction of phytoplankton, however, results obtained by Vidoudez [15] with a wider size spectrum of phytoplankton denotes that small PUA producers can be also important in nature. This is a crucial question to consider when the role of PUAs in natural community interaction is addressed. Cell size is a key parameter in the study of competition for nutrient resources and larger organisms (lower growth rates and higher nutrient requirements) could use these PUAs to outcompete small ones (higher growth rates and lower nutrient requirements). Our results, together with that presented by Bartual *et al*. [32] for open oceanic areas, are the first evidence that physiological stress linked with nutrient availability can modulate the *p*PUA distribution in natural non-blooming areas.

## 3. Experimental Section

### 3.1. Field Sampling

**Figure 5:**
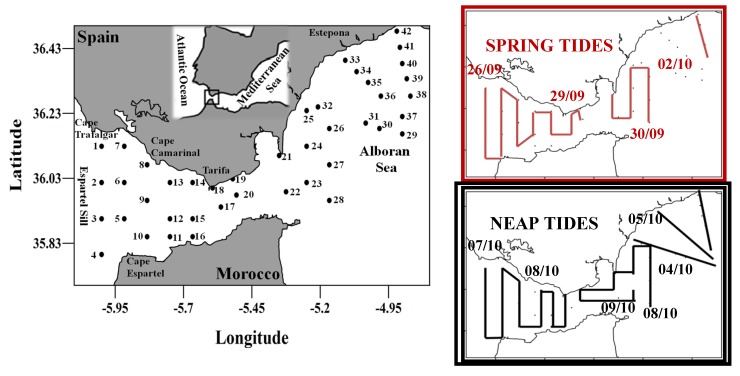
Map showing the location and main geographic points of the study area. Black dots indicate the sampling stations. Track followed by the vessel during Spring Tides and Neap Tides. The start and end time for each survey are also shown.

Data were obtained between 26 September and 9 October of 2008 aboard the research vessel B/O “Sarmiento de Gamboa”. The study area covered the Strait of Gibraltar and the western Alboran Sea (Figure 5), with a total of 42 sampled stations. These stations were sampled twice under two different tidal conditions: spring tides (ST) and neap tides (NT). 25 L of natural seawater were collected at 25 m at each station using Niskin bottles mounted on a rosette sampler. The collected seawater was filtered through two consecutive meshes: a first of 200 µm (to remove the microzooplankton present) and a second of 10 µm, where larger cells of phytoplankton were retained. This phytoplankton was concentrated in 125 ml of 0.7 µm filtered seawater and subsequently filtered through polycarbonate filter with a 0.4 µm pore size (GE Water and Process Technologies, Trevose, PA, USA). The filter was then transferred to 25 mL glass vial (Teknokroma Analítica, S.A., Barcelona, Spain). The cells retained in the filter were rinsed using 1 mL of a 25 mM *O-*(2,3,4,5,6-Pentafluorobenzyl) hydroxylamine hydrochloride (PFBHA, Fluka, Basel, Switzerland) solution in Tris-HCl 100 mM, pH 7.2 [39] and it was stored at −80 °C until further analysis.

### 3.2. Extraction of pPUA

Determination of the production of PUA by the cells was performed according to a modified protocol based on [39]. Once at the lab, when cell suspension was defrost, it was transferred to a conical glass centrifuge tube (VWR International Eurolab, S.L., Barcelona, Spain) and 500 µL of internal standard was then added (benzaldehyde, 10 µM in methanol, Sigma-Aldrich, Buchs, Switzerland). For mechanical disruption of the cells, samples were sonicated by ultrasound (Bandelin Sonoplus, HD2070, 97%) and kept for 1 h at room temperature to ensure derivatization. For extraction, a mixture of water:methanol:hexane (2:1:2) (Methanol for liquid comatography, Licrosolv, 99.8%; Hexane for liquid chromatography, Licrosolv, 98%) were added and the sample was vortexed for 1 min. The mixture was acidified by addition of sulfuric acid (Panreac, 95%–98%) and vortexed again. The hexane upper layer was removed with a glass pasteur pipette with care to prevent contamination from the pipette ball. The hexane was transferred to a 4 mL glass vial (Teknokroma). This extraction was realized twice. The solvent was removed under vacuum. The organic residue, which has remained on wall of the vial was rinsed in 300–500 µL of hexane. The solvent was transferred into 1.5 mL amber glass vials (Waters) and was evaporated under a stream of nitrogen. The vials were closed with caps fitted with PTFE/silicone septa and stored at −80 °C until analysis by GC-MS. Before the analysis and quantification of PUA, the organic residue remaining on the wall was taken up in 50 µL of Hexane (Licrosolv, 98%) and transferred into 0.1 clearglass micro insert (VWR).

### 3.3. Analysis and Quantification of PUA

A Quattro micro GC (Waters^®^ Micromass^®^ Quattro micro™ GC, Milford, MA, USA) tandem quadrupole instrument, coupled to an Agilent 7890A gas chromatograph (GC) (Agilent, Santa Clara, CA, USA) equipped with a DB5-MS 30 m column (0.25 mm internal diameter, 0.25 µm film thickness) was used for GC-MS measurements. Helium 5.0 was the carrier gas with a constant flow of 1 mL min^−1^. The electron energy was 70 eV. The injection was 1 µL in pulse splitless mode, at 250 °C. PUAs were identified by comparison to external standards and the presence of their molecular ions (*m*/*z* 305 for heptadienal, 319 for octadienal and 347 for decadienal) and also the presence of major fragments ions, *i.e.*, *m*/*z* 276 for the PUAs, 271 for the internal standard and 181 for all. Quantification was based on the ratio between the fragment at *m*/*z* 276 of the derivatized PUA and the fragment at *m*/*z* 271 of the derivatized internal standard (that is benzaldehyde). The calibration was performed using synthetic standard of derivatized (25 mM PFBHA-solution in Tris-HCl at pH 7.2) 2*E*,4*E*-heptadienal (90%, Sigma-Aldrich Chemie GmbH, Steinheim, Germany), 2*E*,4*E*-octadienal (≥96%, Sigma-Aldrich Chemie, GmbH, Steinheim, Germany) and 2*E*,4*E*-decadienal (85%, Sigma-Aldrich Chemie, GmbH, Steinheim, Germany) in ranges from 0.5–15 nM, 15–200 nM to 200–8000 nM, to cover the wide range of molarities found in the concentrated extracts of the samples (maximal value of 4200 nM). Calibrations curves were repeated with every group of analyzed samples for three different ranges: 0.5–15 nM, 15–200 nM and 200–8000 nM of each aldehyde. The correlation coefficients obtained for each aldehyde at every range were: *R*^2^ = 0.9884 ± 0.009, 0.9919 ± 0.011 and 0.9963 ± 0.004 for heptadienal; *R*^2^ = 0.9875 ± 0.013, 0.9716 ± 0.037 and 0.9896 ± 0.011 for octadienal; *R*^2^ = 0.9509 ± 0.046, 0.9823 ± 0.005 and 0.9915 ± 0.006 for decadienal, respectively.

Chromatograms were evaluated with the QuanLynx software (version 4.1, Waters, Milford, MA, USA). Identification of the PUA was based on the retention time compared with the standards and the presence of the molecular ion of each derivatized aldehyde. The GC temperature program for the separation was 60 °C (1 min) then increased with a rate of 5 °C min^−1^ to 280 °C and then with a rate of 20 °C min^−1^ to 300 °C (1 min).

### 3.4. Plankton Analysis by Flow-Cytometer and Microscope (FlowCAM)

Particles contained in 5 L of seawater were concentrated on board using a 10 µm mesh, then were fixed (formaldehyde 4%) and stored in dark. Abundance, biovolume and taxonomic composition were analyzed by using a Flow Cytometer and Microscope (FlowCAM^®^, Fluid Imaging Technologies, Yarmouth, ME, USA) [40]. Several stations were selected for microplankton analysis, making a total of 37 samples (19 during ST and 18 during NT). Concentrated samples were divided into two fractions with a 100 µm mesh. Both of them were analyzed in autoimage mode (20 photographs per second, flow rate over 1 mL/min). Fraction with particles larger than 100 µm was prefiltered with a 250 µm mesh to avoid obstructions and used for analysis at ×40 magnification (×4 objective, flowcell 300 µm). The remaining fraction (10–100 µm) was analyzed at ×100 magnification (×10 objective, flowcell 100 µm). All samples were pumped through the FlowCAM at least during 30 min, after that time, if the number of cells counted was under 400 (10% counting error, [41]) the sample continued being measured till it was complete. However, in some samples for spring (ST18, ST20, ST28) and neap tide (ST9, ST11, ST18, ST20, ST, 28, ST42, ST44, ST52) phytoplankton counts have finally been estimated with a 20% counting error (100 cells counted at least).

Images were analyzed by using VisualSpreadsheet® Particle Analyses software 2.4.6 (Fluid Imaging Technologies, Yarmouth, ME, USA). Invalid vignettes (bubbles, detritus and duplicated images) were firstly removed from the database through visual recognition. After that, plankton images were classified in eight plankton groups: five phytoplankton groups (diatoms, dinoflagellates, coccolitophorids, silicoflagellates and “other phytoplankters” for deteriorated phytoplankters or not matching previous groups as cyanophyta) and two zooplankton groups (including copepods and tintinnids) and a final group named “Others” with those plankters not matching previus categories. The sorting was carried out with VisualSpreadSheet automatic classification (based in images libraries: see Supplementary Information). Each group was analyzed after automatic classification and corrected by visual inspection, placing wrong vignettes manually into their proper groups (see Supplementary Information as examples). As diatoms are the main group concerning this study we detailed their classification into six more groups (Table 8) composed by those particles with similar colors, shape and size, and detailing as much as possible the taxonomic composition.

**Table 8:** Diatoms groups and criteria used for classification in this work by FlowCAM methodology.

Group	Shape	Colours	Description and examples
Centric single diatoms	Round	Green/Brown	Centric single diatom cells round in their valvar view. Mainly *Thalassiosira* and *Coscinodiscus* species.
Pennate diatoms	Oval	Green/Brown	e.g., *Pleurosigma* sp.
Linear chains of small cells	Straight	Green/Brown	Small cells making lineal chains. e.g.,: Some species of *Chaetoceros*, *Skeletonema*, *etc*.
Large individuals and linear chains of large cells	Straight	Light green	Long straight single cells or chains. e.g.,: *Rhizosolenia* and *Proboscia* species.
Helical chains	Helical	Light green	Species making helical chains. e.g.,: *Guinardia striata*, *Chaetoceros debilis*.
Other diatoms	−	−	Those diatoms not fitting in previous described groups. Always representing less than 8% in abundance. e.g., *Thalassionema nitzschioides*, *Guinardia flaccida*, *Odontella* sp.

Additionally, the FlowCAM software (Fluid Imaging Technologies, Yarmouth, ME, USA) provided an estimation of cell volume for each cell. It is calculated using the equivalent spherical diameter (ESD) considering all the cells spherical shaped. Such assumption can bias the biomass estimation when particles are cylindrical (most of diatoms) or photographed in different orientations [42] overestimating their volume. So, we used the aspect ratio for each cell (major to minor axis ratio also given by the software) to correct the spherical volume into ellipsoidal volume: Ellipsoidal Volume = Spherical Volume × Aspect Ratio^1/2^ [43].

### 3.5. Biological Variables

Total chlorophyll was estimated from 0.5 L seawater samples filtered through Whatman GF/F filters using the fluorimetric method describe by [44] and modified by [45]. For fractionated chlorophyll estimation (FChla > 20 µm), five liters of seawater were filtered through a nylon mesh with a 20-µm nominal pore size. The fraction retained on the mesh was then collected by washing it with clean, filtered (Whatman GF/F, 0.7 mm) seawater. This fraction was filtered again through a Whatman GF/F filter, and its chlorophyll content was determined following the same fluorimetric protocol described above for total chlorophyll.

The percentage of active chlorophyll (AChla) was estimated using a Pulse Amplitude Modulated (PAM) fluorometer specifically designed to study phytoplankton cells (PhytoPAM^®^, Heinz Walz GmbH, Effeltrich, Germany, see [46] for a detailed description). The PhytoPAM uses weak probe flashes to measure the change in the quantum yield of fluorescence induced by a strong pump flash. This relative change is proportional to the quantity of chlorophyll included in active photosystem II (PS II) reaction centers as successive light pulse leads to a saturation of PS II centers and a diminution of the fluorescence quantum yield. Thus the PhytoPAM provides an estimate of the proportion of total chlorophyll within active PS II, *i.e.*, the chlorophyll available for photosynthesis [47] or “active chlorophyll”. The measurements were done on board with dark-adapted seawater samples from each station and depth as in [22,48].

### 3.6. Nutrients

For nutrients analyses (Dissolved nitrate, phosphate and silicate) three replicates of filtered seawater (12 mL, Whatman GF/F filters) were collected at each sampling point and depth and stored at −20 °C. Nutrient concentrations were measured in the laboratory using an autoanalyzer (Technicon AA-II-TRACS 800) following the techniques of [49].

### 3.7. Statistics

All statistical tests were performed using the program R [38]. Data distributions were tested for normality and heterocedasticity using the Shapiro-Wilk test (*p*-value > 0.05) and Levene’s test (*p*-value > 0.05), respectively. A one-way analysis of variance (ANOVA) followed by Bonferroni’s test was used to test for a significant difference between the two tidal cycles (spring and neap tides). Data were logarithmic transformed prior to analysis.

Principal component analysis (PCA) was used for dividing the data set into homogeneous groups, using the Pearson correlation’s coefficient matrix. This method reduces the data dimensionality by performing a correlation analysis between factors. The procedure transforms a number of possibly correlated variables into a smaller number of uncorrelated factors called principal components. The scores of each variable on the first and second component (axis) were used to examine the variability of the *p*PUAs and PUAs per cells. The variables considered has been % Active chlorophyll, diatom cellular densities, dinoflagellates cellular densities, grazers cellular densities, diatom cellular biovolume, dinoflagellates cellular biovolume, grazers cellular biovolume, silicate, nitrate, and phosphate. The *p*PUAs and diatom spatial distribution was produced by interpolation using the krigging method in the Surfer software (Golden Software, v8.05, Inc., Golden, CO, USA).

## 4. Conclusions

In this work, we show a significant presence of PUA producer diatoms in the Strait of Gibraltar at different tidal cycles, and coastal areas with permanently high abundance of these producers can be found. These natural assemblages dominated by diatoms would produce mainly decadienal, followed by heptadienal and octadienal. The relevance of decadienal contribution to total *p*PUA is an interesting result since this aldehyde is not usually considered as a typical diatom metabolite. Differences in PUA spectra of isolated diatoms *vs*. natural assemblages should be prospected. By first time we found that physiologically stressed large size diatoms could release higher amounts of PUAs in nature, modulated by nutrient availability in natural non-blooming areas.

## Supplementary Files

Supplementary File 1 Supplementary Information (PDF, 941 KB)

## References

[1] Smetacek V. (2001). A watery arms race. Nature.

[2] Wilhelm C., Büchel C., Fisahn J., Goss R., Jakob T., LaRoche J., Lavaud J., Lohr M., Riebesell U., Stehfest K. (2006). The regulation of carbon and nutrient assimilation in diatoms is significantly different from green algae. Protist.

[3] Hamm C.E., Merkel R., Springer O., Jurkojc P., Maier C., Prechtel K., Smetacek V. (2003). Architecture and material properties of diatom shells provide effective mechanical protection. Nature.

[4] Miralto A., Barone G., Romano G., Poulet S.A., Ianora A., Russo G.L., Buttino I., Mazzarella G., Laabir M., Cabrini M. (1999). The insidious effect of diatoms on copepod reproduction. Nature.

[5] Pohnert G. (2005). Diatom/Copepod interactions in plankton: The indirect chemical defense of unicellular algae. ChemBioChem.

[6] Pohnert G., Schulz S. (2004). Chemical Defense Strategies of Marine Organisms. Chemistry of Pheromones and Other Semiochemicals I.

[7] Vardi A., Formiggini F., Casotti R., de Martino A., Ribalet F., Miralto A., Bowler C. (2006). A stress surveillance system based on calcium and nitric oxide in marine diatoms. PLoS Biol..

[8] Fontana A., d’Ippolito G., Cutignano A., Miralto A., Ianora A., Romano G., Cimino G. (2007). Chemistry of oxylipin pathways in marine diatoms. Pure Appl. Chem..

[9] D’Ippolito G., Tucci S., Cutignano A., Romano G., Cimino G., Miralto A., Fontana A. (2004). The role of complex lipids in the synthesis of bioactive aldehydes of the marine diatom *Skeletonema costatum*. Biochim. Biophys. Acta.

[10] D’Ippolito G., Iadicicco I., Romano G., Fontana A. (2002). Detection of short-chain aldehydes in marine organism: The diatom *Thalassiosira rotula*. Tetrahedron Lett..

[11] Pohnert G. (2002). Phospholipase A_2_ activity triggers the wound-activated chemical defense in the diatom *Thalassiosira rotula*. Plant Physiol..

[12] Ribalet F., Wichard T., Pohnert G., Ianora A., Miralto A., Casotti R. (2007). Age and nutrient limitation enhance polyunsaturated aldehyde production in marine diatoms. Phytochemistry.

[13] Ribalet F., Vidoudez C., Cassin D., Pohnert G., Ianora A., Miralto A., Casotti R. (2009). High plasticity in the production of diatom-derived polyunsaturated aldehydes under nutrient limitation: Physiological and ecological implications. Protist.

[14] Vidoudez C., Pohnert G. (2008). Growth phase-specific release of polyunsaturated aldehydes by the diatom *Skeletonema marinoi*. J. Plankton Res..

[15] Vidoudez C., Casotti R., Bastianini M., Pohnert G. (2011). Quantification of dissolved and particulate polyunsaturated aldehydes in the Adriatic Sea. Mar. Drugs.

[16] Gómez F., Echevarría F., García C.M., Prieto L., Ruiz J., Reul A., Jiménez-Gómez F., Varela M. (2000). Microplankton distribution in the Strait of Gibraltar: Coupling between organisms and hydrodynamic structures. J. Plankton Res..

[17] Lacombe H., Richez C., Nihoul J.C.J. (1982). The Regime of the Strait of Gibraltar. Hydrodynamics of Semi-Enclosed Seas.

[18] Gascard J.C., Richez C. (1985). Water masses and circulation in the Western Alboran Sea and in the Straits of Gibraltar. Prog. Oceanogr..

[19] Macías D., Martin A.P., García-Lafuente J., García C.M., Yool A., Bruno M., Vázquez-Escobar A., Izquierdo A., Sein D.V., Echevarría F. (2007). Analysis of mixing and biogeochemical effects induced by tides on the Atlantic-Mediterranean flow in the Strait of Gibraltar through a physical-biological coupled model. Prog. Oceanogr..

[20] Ramírez-Romero E., Macías D., Bruno M., Reyes E., Navarro G., García C.M. (2012). Submesoscale, tidally-induced biogeochemical patterns in the Strait of Gibraltar. Estuar. Coast. Shelf Sci..

[21] Macías D., García C.M., Echevarría F., Vázquez-López A., Bruno M. (2006). Tidal induced variability of mixing processes on Camarinal Sill (Strait of Gibraltar): A pulsating event. J. Mar. Syst..

[22] Macías D., Lubían L.M., Echevarría F., Huertas I.E., García C.M. (2008). Chlorophyll maxima and water mass interfaces: Tidally induced dynamics in the Strait of Gibraltar. Deep-Sea Res. Part I: Oceanogr. Res..

[23] Vidoudez C., Nejstgaard J.C., Jakobsen H.H., Pohnert G. (2011). Dynamics of dissolved and particulate polyunsaturated aldehydes in mesocosms inoculated with different densities of the diatom *Skeletonema marinoi*. Mar. Drugs.

[24] Gómez F., González N., Echevarría F., García C.M. (2000). Distribution and fluxes of dissolved nutrients in the strait of Gibraltar and its relationships to microplankton biomass. Estuar. Coast. Shelf Sci..

[25] Redfield A.C., Ketchum B.H., Richards F.A., Hill M.N. (1963). The Influence of Organisms on the Composition of Seawater. The Composition of Sea-Water Comparative and Descriptive Oceanography.

[26] Smayda T.J. (1997). Harmful algal bloom: Their ecophysiology and general relevance to phytoplankton blooms in the sea. Limnol. Oceanogr..

[27] Goering J.J., Nelson D.M., Carter J.A. (1973). Silicic acid uptake by natural populations of marine phytoplankton. Deep-Sea Res..

[28] Paasche E. (1980). Silicon content of five marine plankton diatom species measured with a rapid filter method. Limnol. Oceanogr..

[29] Wichard T., Poulet S.A., Halsband-Lenk C., Albaina A., Harris R., Liu D., Pohnert G. (2005). Survey of the chemical defense potential of diatoms: Screening of 51 species for α,β,λ,δ-unsaturated aldehydes. J. Chem. Ecol..

[30] Wichard T., Poulet S.A., Boulesteix A.L., Ledoux J.B., Lebreton B., Marchetti J., Pohnert G. (2008). Influence of diatoms on copepod reproduction. II. Uncorrelated effects of diatom-derived α,β,λ,δ-unsaturated aldehydes and polyunsaturated fatty acids on *Calanus helgolandicus* in the field. Prog. Oceanogr..

[31] Hansen E., Ernstsen A., Eilertsen H.C. (2004). Isolation and characterisation of a cytotoxic polyunsaturated aldehyde from the marine phytoplankter Phaeocystis pouchetii (Hariot) Lagerheim. Toxycology.

[32] Bartual A., Arandia-Gorostidi N., Cózar A., Morillo-García S., Ortega M.J., Vidal M., Cabello A.M., González-Gordillo J.I., Echevarría F. (2014). Polyunsaturated aldehydes from large phytoplankton of the Atlantic Ocean (42°N to 33°S). Mar. Drugs.

[33] Vargas-Yáñez M., Viola T.S., Jorge F.P., Rubin J.P., García-Martínez M.C. (2002). The influence of tide-topography interaction on low-frequency heat and nutrient fluxes. Application to Cape Trafalgar. Cont. Shelf Res..

[34] Navarro G., Ruiz J. (2006). Spatial and temporal variability of phytoplankton in the Gulf of Cádiz through remote sensing images. Deep Sea Res. Part II: Top. Stud. Oceanogr..

[35] Groth-Nard C., Robert J.M. (1993). Les lipids des diatomees. Diatom Res..

[36] Legrand C., Rengefors K., Fistarol G.O., Granéli E. (2003). llelopathy in phytoplankton-biochemical, ecological and evolutionary aspects. Phycologia.

[37] Vidoudez C., Pohnert G. (2012). Comparative metabolomics of the diatom Skeletonema marinoi in different growth phases. Metabolomics.

[38] R Development Core Team (2008). R: A Language and Environment for Statistical Computing. R Foundation for Statistical Computing, Vienna, Austria. ISBN 3-900051-07-0. http://www.R-project.org.

[39] Wichard T., Poulet S.A., Pohnert G. (2005). Determination and quantification of α,β,λ,δ-unsaturated aldehydes as pentafluorobenzyl-oxime derivates in diatom cultures and natural phytoplankton populations: Application in marine field studies. J. Chromatogr. B.

[40] Sieracki C.K., Sieracki M.E., Yentsch C.S. (1998). An imaging-in-flow system for automated analysis of marine microplankton. Mar. Ecol. Prog. Ser..

[41] Lund J.W.G., Kipling C., Le Cren E.D. (1958). The inverted microscope method of estimating algal numbers and the statistical basis of estimations by counting. Hydrobiologia.

[42] Álvarez E., López-Urrutia A., Nogueira E. (2012). Improvement of plankton biovolume estimates derived from image-based automatic sampling devices: Application to FlowCAM. J. Plankton Res..

[43] Herman A. (2009). LOPC Data Analyses-Standard LOPC. http://www.alexherman.com/lopc_post.php.

[44] Yentsch C.S., Menzel D.W. (1963). A method for the determination of phytoplankton chlorophyll and Phaeophytin by fluorescence. Deep-Sea Res..

[45] Holm-Hassen O., Lorenzen C.J., Homes R.W., Strickland J.D.H. (1965). Fluorometric determination of chlorophyll. J. Cons. Perm. Int. Explor. Mer.

[46] Kolbowski J., Schreiber U., Mathis V.P. (1995). Computer-Controlled Phytoplankton Analyzer Based on a 4-Wavelengths PAM Chlorophyll Fluorometer. Photosynthesis, from Light to Biosphere.

[47] Kolberg Z., Falkowski P.G. (1993). Use of active fluorescence to estimate phytoplankton photosynthesis *in situ*. Limnol. Oceanogr..

[48] Macías D., Navarro G., Bartual A., Echevarría F., Huertas I.E. (2009). Primary production in the Strait of Gibraltar: Carbon fixation rates in relation to hydrodynamic and phytoplankton dynamics. Estuar. Coast. Shelf Sci..

[49] Strickland J.D.H., Parsons T.R. (1972). A Practical Handbook of Seawater Analysis.

