# Supplementary material for: Potential Polyunsaturated Aldehydes in the Strait of Gibraltar under Two Tidal Regimes
Source: Mar Drugs. 2014 Mar 13;12(3):1438–59. doi: 10.3390/md12031438 (PMC3967220; doi:10.3390/md12031438)
Supplement: Supplementary File 1 — Supplementary Information (PDF, 941 KB) [file marinedrugs-12-01438-s001.pdf]

Supplementary Information

Table S1. Results by sampling site obtained during Spring Tide regime. Data account for the sampling sites where plankton community was analyzed by Flow CAM methodology.

	**Spring Tides**																	
**Stations**	**1**	**3**	**5**	**6**	**8**	**9**	**10**	**12**	**13**	**14**	**17**	**23**	**24**	**25**	**26**	**27**	**28**	**41**
**Diatom cell densities ($\times 10^3$ cell L^{-1}) and biovolume ($\times 10^6$ μm^3 L^{-1})**																		
**Abundance**	50.9	2.0	0.7	9.5	1.7	2.0	0.5	0.7	2.6	10.2	0.3	5.9	0.7	7.6	1.5	1.6	5.1	0.9
**Biovolume**	6,350,626	88.3	71.8	1,428,711	71.3	324.4	8.5	8.4	89.0	2,298,395	9.0	211,611	25.8	513.0	89.2	121.4	619.3	102.3
**% Microplanktonic community (abundance)**																		
**Diatoms**	95.9	63.9	68.5	87.2	79.7	80.7	54.6	51.1	89.4	82.2	54.8	93.8	84.3	88.9	69.9	66.5	89.3	68.8
**Dinoflagellates**	2.0	8.7	20.5	9.5	16.2	12.3	21.0	11.3	5.4	3.6	24.9	5.0	10.7	8.1	18.6	19.6	7.8	23.8
**Grazers**	0.5	0.3	1.4	1.1	2.4	1.2	1.9	1.8	0.0	0.3	4.5	0.4	2.2	0.3	1.7	0.5	1.1	4.7
**% Microplanktonic community (biovolume)**																		
**Diatoms**	99.6	85.7	65.7	99.9	28.6	72.8	30.8	19.5	74.3	99.9	20.6	99.9	51.5	73.5	46.7	54.9	87.2	50.3
**Dinoflagellates**	0.3	8.3	12.9	0.0	42.8	16.0	7.0	11.7	13.5	0.0	28.3	0.0	34.3	10.7	25.0	16.9	2.9	13.9
**Grazers**	0.00	2.2	3.8	0.0	28.3	10.0	39.9	22.3	5.5	0.0	46.9	0.0	10.5	12.9	23.8	22.3	7.5	25.3
***p*PUAs from cells from 1 L**																		
**HD**	1.9	0.2	0.5	0.9	0.9	1.05	1.4	0.3	0.3	0.4	0.1	0.5	0.02	0.03	0.8	0.5	0.8	0.02
**OD**	3.1	0.01	0.00	1.1	0.2	0.2	1.2	0.02	0.1	0.2	0.00	1.2	0.1	0.05	0.00	0.06	0.2	0.005
**DD**	6.3	0.09	0.4	1.5	1.9	1.6	2.4	1.4	1.1	1.2	0.01	1.8	1.6	1.4	1.4	1.6	1.5	0.02
**Total PUAs**	11.4	0.3	0.9	3.7	3.2	3.0	5.0	1.7	1.6	1.8	0.1	3.6	1.8	1.5	2.3	2.2	2.6	0.05
**PUAs per cells (fmol $cell^{-1}$)**																		
**HD**	0.03	0.1	0.6	0.1	0.5	0.5	2.7	0.4	0.1	0.04	0.3	0.09	0.03	0.004	0.5	0.3	0.1	0.02
**OD**	0.06	0.006	0.01	0.1	0.1	0.1	2.3	0.03	0.04	0.02	0.001	0.2	0.2	0.007	0.003	0.03	0.04	0.005
**DD**	0.1	0.04	0.5	0.1	1.1	0.8	4.7	1.9	0.4	0.1	0.05	0.3	2.0	0.1	0.9	1.0	0.2	0.02
**Total PUAs**	0.2	0.1	1.2	0.3	1.7	1.4	9.8	2.3	0.6	0.1	0.3	0.6	2.3	0.2	1.5	1.3	0.5	0.05

Table S1. *Cont.*

	**Spring Tides**																	
**Stations**	**1**	**3**	**5**	**6**	**8**	**9**	**10**	**12**	**13**	**14**	**17**	**23**	**24**	**25**	**26**	**27**	**28**	**41**
	**Chlorophyll (µg L^{-1})**																	
**Chla > 20 µm**	0	0.02	0	0.3	0.06	0.04	0.01	0.01	0.1	0.2	0.04	0.3	0.07	0.2	0.04	0.09	0.2	0.07
**Chla Total**	4.2	0.1	0.4	2.1	0.5	0.2	0.4	0.3	0.9	3.9	0.4	2.3	1.0	1.5	1.1	1.3	1.4	1.0
**% Active Chlorophyll**	65.0	0	0	54.9	19.8	0	22.2	17.3	30.7	49.6	0	46.6	17.1	22.1	23.6	18.3	34.6	27.3
	**Nutrients (µM)**																	
**Nitrate**	0.8	0.5	2.5	1.2	3.8	0.2	1.0	3.1	2.1	2.4	-	2.2	1.4	0.9	0.4	0.2	1.6	1.7
**Phosphate**	nd	0.09	nd	nd	nd	nd	nd	nd	nd	nd	-	nd	nd	nd	nd	0.3	0.1	nd
**Silicate**	1.5	1.0	4.6	3.5	3.0	2.8	2.2	1.9	3.3	3.7	-	1.2	1.0	0.7	0.8	1.1	2.0	1.7

Table S2. Results by sampling site obtained during Neap Tide regime. Data account for the sampling sites where plankton community was analyzed by Flow CAM methodology.

	**Neap Tides**														
**Stations**	**1**	**5**	**6**	**8**	**9**	**10**	**14**	**19**	**23**	**24**	**25**	**26**	**28**	**36**	**41**
	**Diatom cell densities (×10^3 cell L^{-1}) and biovolume (×10^6 µm^3 L^{-1})**														
**Abundance**	1.1	0.3	5.9	6.8	1.2	0.4	11.3	5.6	1.1	0.9	1.8	5.1	0.6	0.2	1.0
**Biovolume**	26.4	2.1	996.7	952.1	86.2	14.6	784.5	186.6	40.7	22.7	162.8	867.4	12.7	6.9	250.0
	**% Microplanktonic community (abundance)**														
**Diatoms**	61.6	44.7	86.5	89.0	62.9	68.8	89.7	86.1	72.0	79.3	66.7	90.8	66.7	34.6	78.3
**Dinoflagellates**	25.2	22.3	9.4	9.5	27.3	23.1	7.7	11.9	21.3	16.1	29.5	8.0	18.0	59.7	12.5
**Grazers**	1.4	13.4	2.0	0.9	4.2	1.1	0.5	0.5	3.9	2.2	1.0	0.3	3.3	4.1	4.6
	**% Microplanktonic community (biovolume)**														
**Diatoms**	28.1	8.7	87.7	88.4	35.3	28.6	84.6	63.8	33.5	38.5	40.2	93.0	26.5	7.7	68.0
**Dinoflagellates**	20.9	49.9	2.4	3.5	16.3	19.2	4.9	16.3	24.1	11.5	27.6	1.9	15.0	44.6	10.9
**Grazers**	31.4	31.0	8.9	5.4	41.0	29.1	8.9	17.1	35.3	39.7	27.6	1.4	52.5	31.6	16.6

Table S2. *Cont.*

**Neap Tides**															
**Stations**	**1**	**5**	**6**	**8**	**9**	**10**	**14**	**19**	**23**	**24**	**25**	**26**	**28**	**36**	**41**
***p*PUAs from cells from 1 L**															
**HD**	1.1	0.03	1.6	0.8	0.5	0.4	0.9	1.4	0.002	0	1.5	3.1	0.01	1.2	1.1
**OD**	1.1	0.001	1.5	0.02	0.02	0.000	0.3	0.04	0.02	0.01	0.3	1.7	0.02	0.1	1.3
**DD**	3.0	0.01	5.7	1.0	1.2	0.01	1.9	2.0	0.3	0.5	4.9	4.9	0.5	0.3	2.4
**Total PUAs**	5.4	0.04	8.8	1.8	1.8	0.4	3.2	3.5	0.3	0.5	6.8	9.8	0.5	1.7	5.0
**PUAs per cells (fmol cell^{-1})**															
**HD**	0.9	0.08	0.2	0.1	0.4	0.9	0.08	0.2	0.002	0.006	0.8	0.5	0.01	5.3	1.1
**OD**	1.0	0.003	0.2	0.004	0.02	0.002	0.03	0.007	0.01	0.01	0.1	0.3	0.04	0.7	1.3
**DD**	2.6	0.04	0.9	0.1	0.9	0.03	0.1	0.3	0.3	0.6	2.6	0.9	0.8	1.3	2.4
**Total PUAs**	4.6	0.1	1.4	0.2	1.4	0.9	0.2	0.6	0.3	0.6	3.6	1.8	0.9	7.3	4.8
**Chlorophyll (µg L^{-1})**															
**Chla > 20 µm**	0.03	0.002	0.2	0.1	0.05	0.03	0.1	0.2	0.1	0.04	0.1	0.2	0	0.01	0.2
**Chla Total**	0.6	0.2	0.6	1.2	0.9	0.7	0.5	0.9	0.9	0.5	1.1	1.8	0.5	0.2	0.6
**% Active Chlorophyll**	0	0	36.3	31.1	0	20.1	0	0	14.3	0	0	27.7	0	0	0
**Nutrients (µM)**															
**Nitrate**	0.1	0	0	0.8	0	0.09	1.1	1.9	2.0	0	0.05	1.3	1.0	0	0.09
**Phosphate**	nd	nd	nd	nd	nd	0.2	0.04	0.03	0.1	0.08	nd	nd	nd	0.04	nd
**Silicate**	1.7	1.6	0.7	1.1	1.2	1.4	1.6	3.5	2.8	2.9	1.3	3.3	1.6	24.1	5.3

Figure S1. Relation between total and fractionated chlorophyll in the sampled stations during Spring tide (red symbols) and Neap tide (Black symbols) regimes.

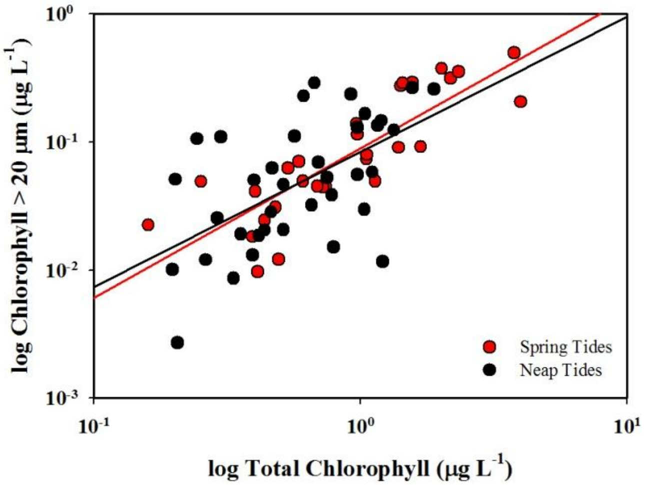

$$[y = 1.16x - 1.05; R^2 = 0.7] \quad (1)$$

$$[y = 1.05x - 1.07; R^2 = 0.3] \quad (2)$$

Main groups used for microplankton classifications. All the images have been selected from the libraries used for the automatic classification. Vignettes presented here were taken with ×40 and ×100 magnification so are not comparable in size terms.

S1. Centrics Single Diatoms

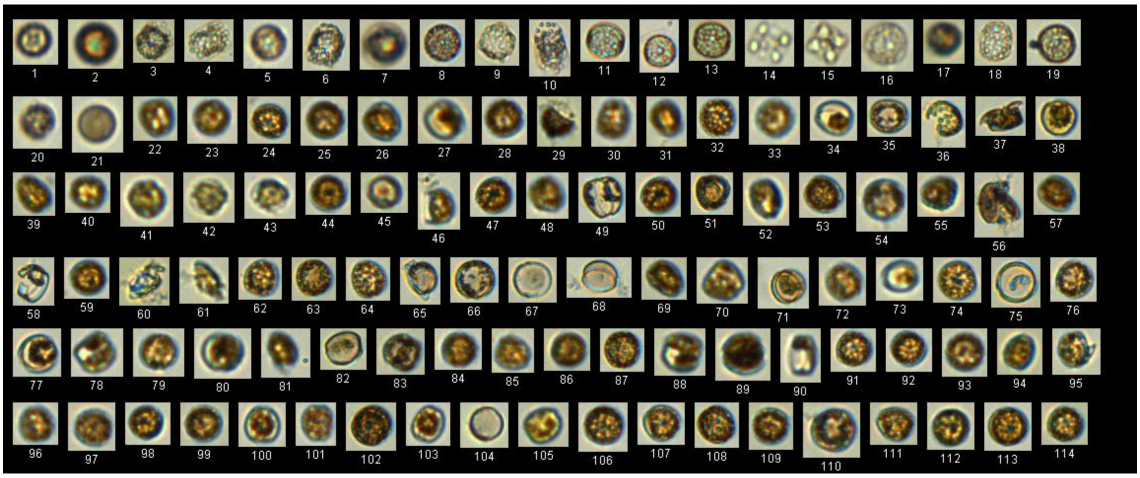

S2. Pennate Diatoms

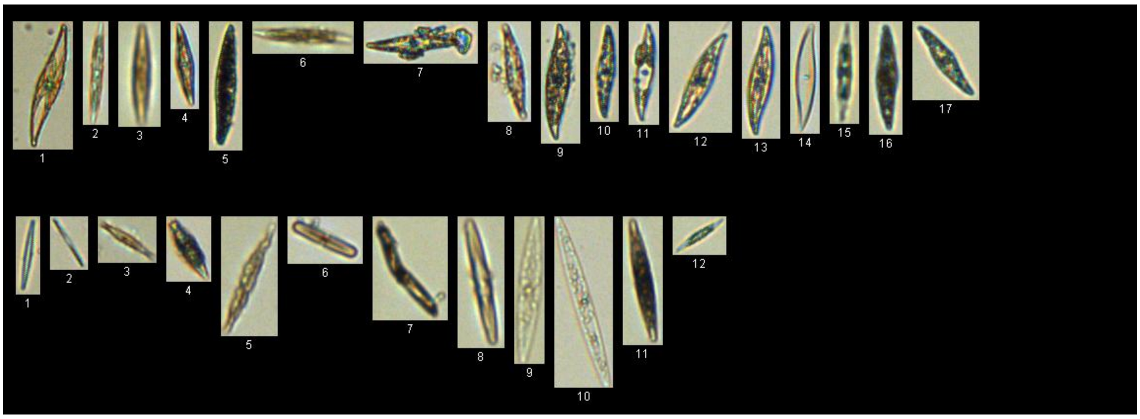

S3. Lineal Small Cells Chains

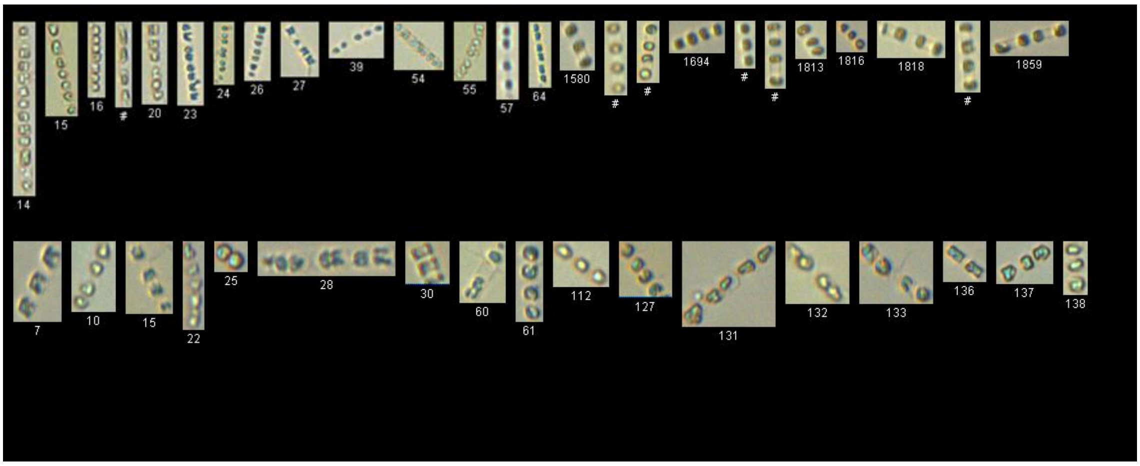

S4. Large Individual and Lineal Chains of Large Cells

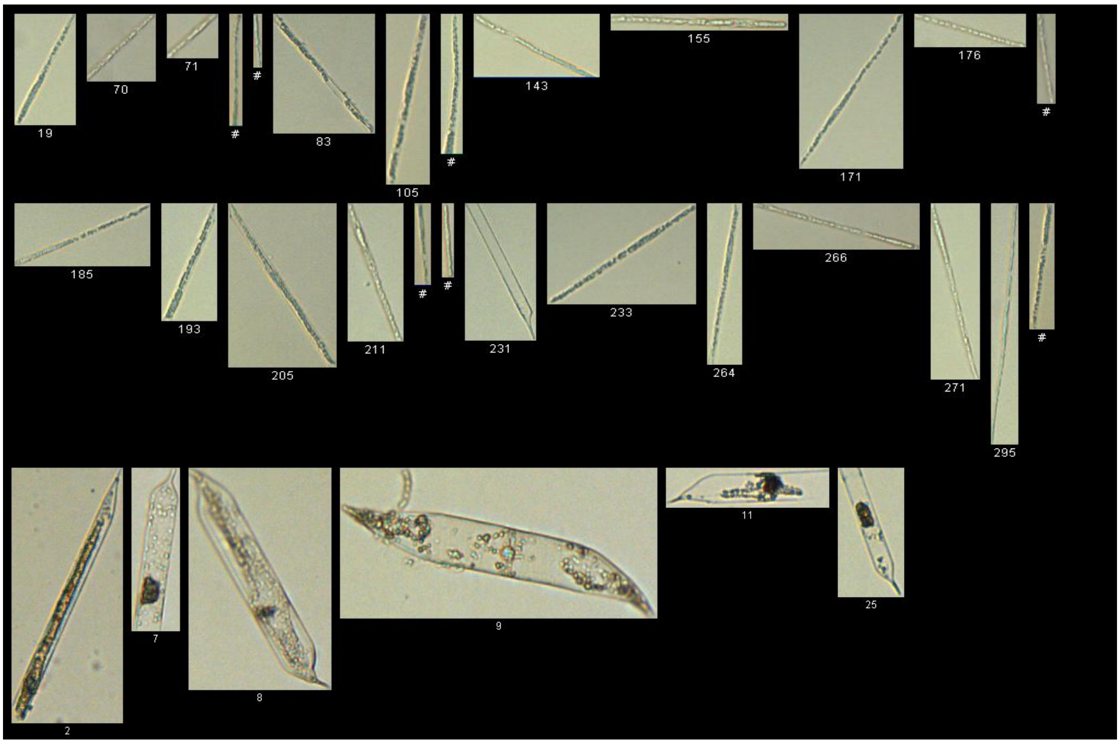

S5. Helical Chains

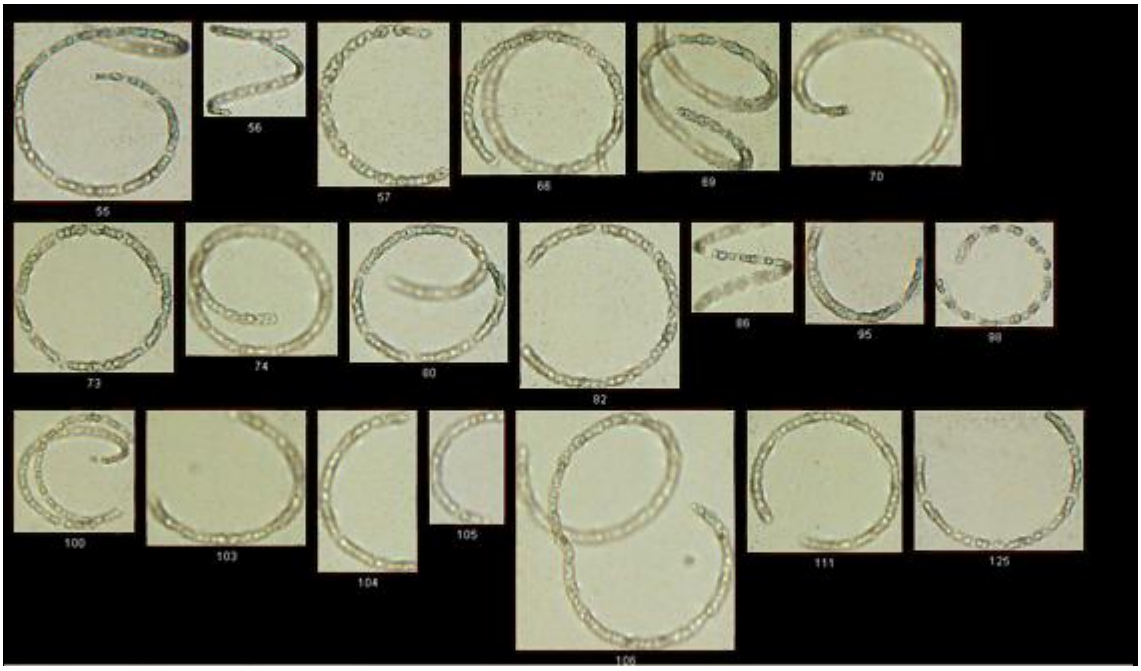

S6. Dinoflagellates

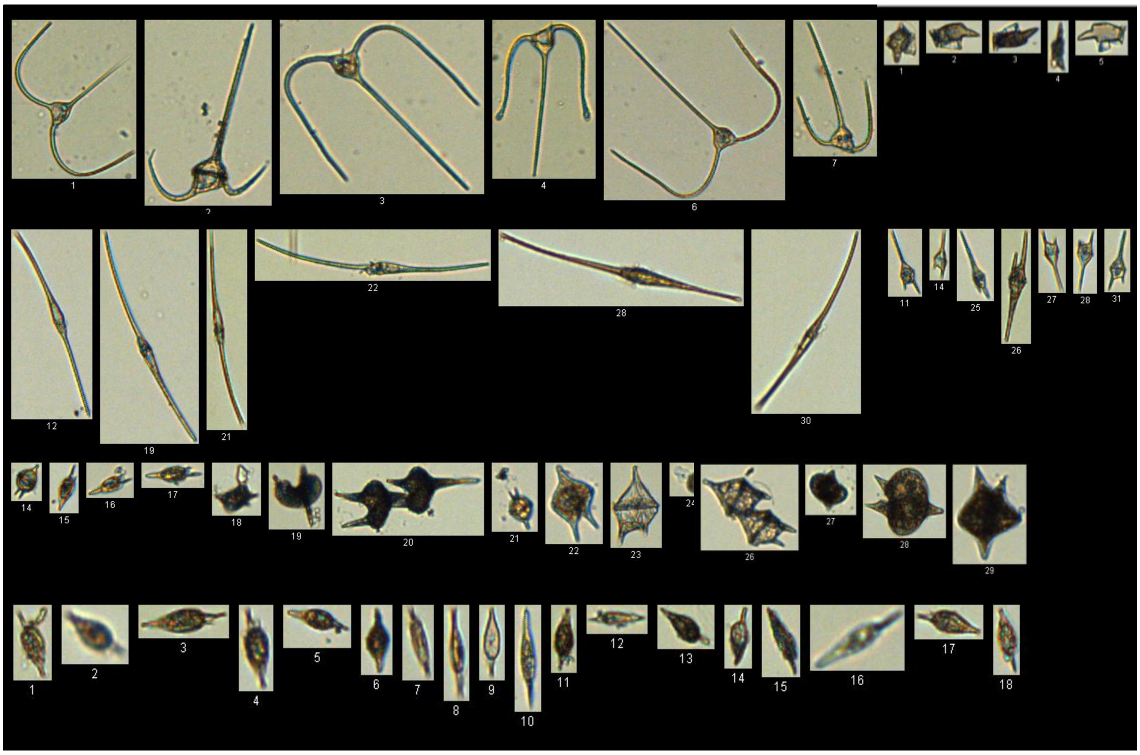

S7. Foraminifera

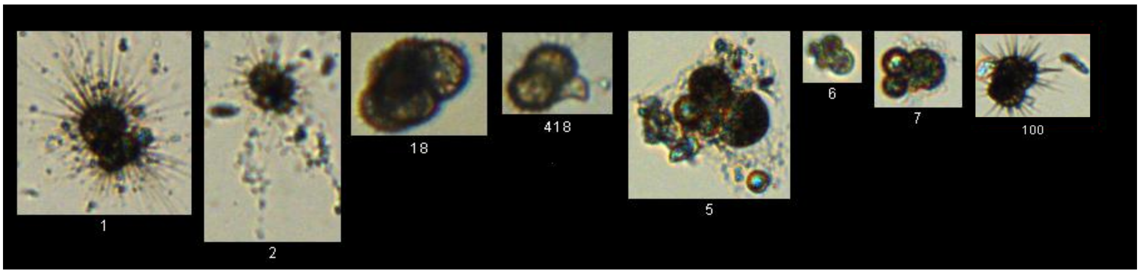

S8. Silicoflagellata

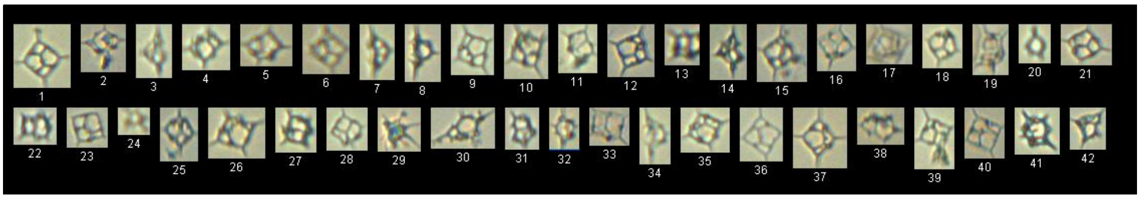

S9. Zooplankton (Copepods + Tintinnids)

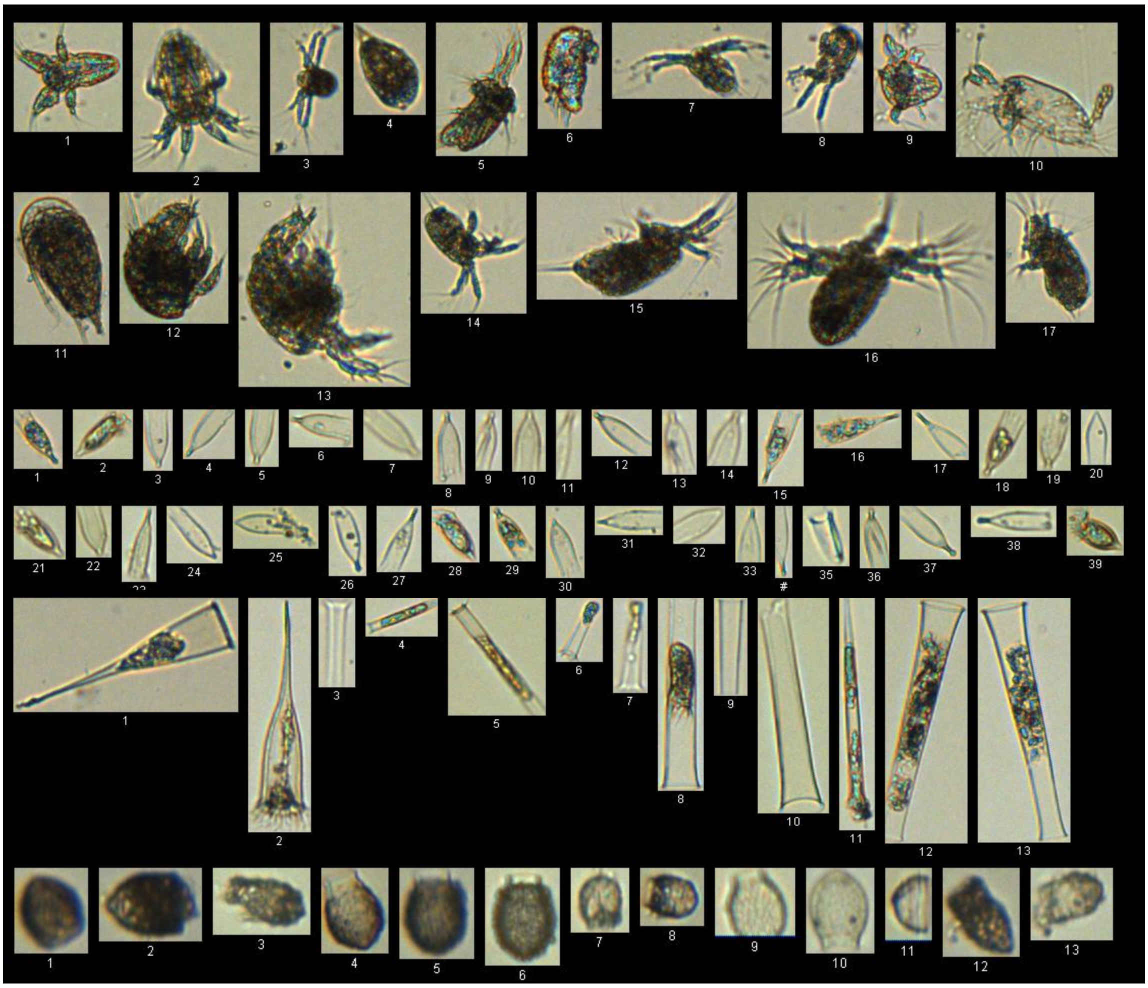

© 2014 by the authors; licensee MDPI, Basel, Switzerland. This article is an open access article distributed under the terms and conditions of the Creative Commons Attribution license (http://creativecommons.org/licenses/by/3.0/).
